# Complete genome of the switchgrass endophyte *Enterobacter clocace* P101

**DOI:** 10.4056/sigs.4808608

**Published:** 2014-02-15

**Authors:** Jodi L. Humann, Mark Wildung, Derek Pouchnik, Austin A. Bates, Jennifer C. Drew, Ursula N. Zipperer, Eric W. Triplett, Dorrie Main, Brenda K. Schroeder

**Affiliations:** 1 Department of Horticulture and Landscape Architecture, Washington State University, Pullman, WA, USA; 2 School of Molecular Biosciences, Washington State University, Pullman, WA, USA; 3 Department of Plant Pathology, Washington State University, Pullman, WA, USA; 4 Department of Microbiology and Cell Science, University of Florida, Gainesville, FL, USA

## Abstract

The *Enterobacter cloacae* complex is genetically very diverse. The increasing number of complete genomic sequences of *E. cloacae* is helping to determine the exact relationship among members of the complex. *E. cloacae* P101 is an endophyte of switchgrass (*Panicum virgatum*) and is closely related to other *E. cloacae* strains isolated from plants. The P101 genome consists of a 5,369,929 bp chromosome. The chromosome has 5,164 protein-coding regions, 100 tRNA sequences, and 8 rRNA operons.

## Introduction

Numerous *Enterobacter cloacae* strains have been associated with plants as agents of disease [1-4], but *E. cloacae* strains have also been associated with plants as endophytes [5-8], used for biocontrol of fungal pathogens [9-16], and associated with nosocomial infections in hospital settings [17-19]. *E. cloacae* is in the *E. cloacae* complex, which also includes the *Enterobacter* species of *E. asburiae*, *E. hormaechei*, *E. kobei*, *E. ludwigii*, and *E. nimipressuralis*. While 16S rRNA sequences are used to initially identify *E. cloacae* strains, the sequence is not always sufficient for identification at the species and sub-species level [17]. Previous phylogenetic studies with multi-locus sequence analyses of common housekeeping genes demonstrate that there is considerable diversity among the strains designated as *E. cloacae* due to the formation of multiple clades and the fact that only 3% of the strains group with the type strain *E. cloacae* subsp. *cloacae* ATCC 13047 [17,18]. The number of draft and complete *E. cloacae* genomes has increased recently and there are currently five complete and five draft *E. cloacae* genomes, with additional registered genome projects [20]. Sequencing and analysis of more *E. cloacae* genomes may establish a basis for explaining the diversity within the *E. cloacae* complex and provide new means for more definitive species or sub-species designation. 

## Classification and features

**Figure 1:**
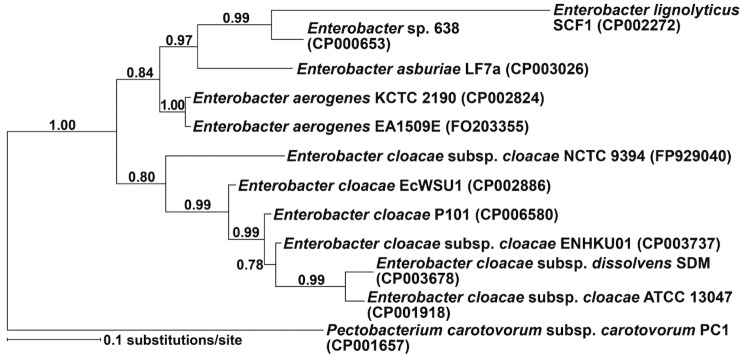
Phylogenetic tree of 16S rRNA sequences from *Enterobacter* sp. with genome sequences. *E. cloacae* strains grouped separately into a clade from other *Enterobacter* species using Bayesian phylogenetic analyses of the 16S rRNA region. Analyses were implemented in MRBAYES [39] and the Bayesian Information Criterion (BIC), DT-ModSel [40] was used to determine the nucleotide substitution model best suited for the dataset. To ensure that the average split frequency between runs was less than 1%, the Markov chain Monte Carlo search included two runs with four chains each for 10,000,000 generations. *Pectobacterium carotovorum* served as the outgroup for the analysis. Numbers in parentheses behind the bacterial names correspond to the GenBank accession numbers for the genome sequences. The scale bar indicates the number of substitutions/site.

*E. cloacae* P101 was isolated from switchgrass (*Panicum virgatum*) growing on Buena Vista Quarry Prairie near Plover, Wisconsin and is a Gram-negative, rod shaped bacterium of the family *Enterobacteriaceae* (Table 1). The species within the genus *Enterobacter* are difficult to identify with biochemical and phylogenetic tests [18], but the increasing number of complete genomes is providing clues as to the relationships among the species. *E. cloacae* species group separately from other *Enterobacter* species in a phylogenetic tree using 16s rRNA sequences (Figure 1) with strong support (posterior probability of 100%). In this analysis, P101 is most closely related to *E. cloacae* EcWSU1 and *E. cloacae* ENHKU01 which are two other *E. cloacae* strains that have been isolated from plants. *E. cloacae* EcWSU1 causes *Enterobacter* bulb decay on stored onions (*Allium cepa*) [41] and *E. cloacae* ENHKU01 was isolated as an endophyte from a pepper (*Capsicum annuum)* plant infected with *Ralstonia solanacearum* [42].

**Table 1:** Classification and general features of *Enterobacter cloacae* P101 according to MIGS recommendations [21]

MIGS ID	Property	Term	Evidence Code
		Domain *Bacteria*	TAS [22]
		Phylum *Proteobacteria*	TAS [23]
		Class *Gammaproteobacteria*	TAS [24-26]
	Current classification	Order *Enterobacteriales*	TAS [27]
		Family *Enterobacteriaceae*	TAS [28-30]
		Genus *Enterobacter*	TAS [19,28,30-33]
		Species *Enterobacter cloacae*	TAS [19,28,32]
		Strain P101	TAS [34-36]
	Gram stain	negative	TAS [37]
	Cell shape	rod	TAS [37]
	Motility	motile via peritrichous flagella	TAS [37]
	Sporulation	non-sporulating	TAS [37]
	Temperature range	mesophilic, 25-40°C	TAS [37]
	Optimum temperature	30-37°C	TAS [37]
	Salinity	not reported	
MIGS-22	Oxygen requirement	facultative anaerobe	TAS [37]
	Carbon source	carbohydrates	TAS [37]
	Energy source	chemoorganotroph	TAS [37]
MIGS-6	Habitat	soil, switchgrass	TAS [34,35]
MIGS-15	Biotic relationship	free-living	TAS [37]
MIGS-14	Pathogenicity	pathogenic on onion bulb	IDA
	Biosafety level	2	
	Isolation	Isolated from switchgrass	TAS [35]
MIGS-4	Geographic location	Wisconsin, USA	TAS [35]
MIGS-5	Sample collection time	not reported	

Evidence codes – IDA: Inferred from Direct Assay (first time in publication); TAS: Traceable Author Statement (i.e., a direct report exists in the literature); NAS: Non-traceable Author Statement (i.e., not directly observed for the living, isolated sample, but based on a generally accepted property for the species, or anecdotal evidence). These evidence codes are from the Gene Ontology project [38]. If the evidence code is IDA, then the property was directly observed for a live isolate by one of the authors, or an expert mentioned in the acknowledgements.

## Genome sequencing and annotation

### Genome project history

The *E. cloacae* P101 genome project was initiated as part of an undergraduate class at the University of Florida [36]. For the class, whole-genome sequence was obtained using a Genome Sequencer 20 (454 Life Sciences, Branford, CT) and the students used PCR and sequencing to resolve some gaps. Although the project began with these data, little progress was made towards closing the genome. As a result, new next-generation DNA sequencing data for P101 was obtained at the Laboratory for Biotechnology and Bioanalysis at Washington State University using the PacBio RS platform and the PCR products generated to confirm the genome assembly were sequenced at Elim Biopharmaceuticals (Hayward, CA). A *Bgl*II cut optical map of P101 was obtained from OpGen (Gaithersburg, MD) in 2009 and was also used in the genome assembly process. The complete chromosome sequence has been deposited in GenBank under the accession number CP006580. Table 2 summarizes the P101 sequencing project.

**Table 2:** P101 Genome sequencing project information

MIGS ID	Property	Term
MIGS-29	Sequencing platform	PacBio RS
MIGS-31	Finishing quality	Finished
MIGS-31.2	Fold coverage	130×
MIGS-30	Assembler	HGAP [43] protocol, SMRT Analysis 2.0.0
MIGS-32	Gene calling method	NCBI Prokaryotic Genome Annotation Pipeline [44]
	GenBank ID	CP006580
	GenBank date of release	December 31, 2013
	Project relevance	Plant-microbe interactions

### Growth conditions and DNA isolation

*E. cloacae* P101 was cultured overnight in LB broth [45] on a rotary shaker at 200 rpm at 28°C. To remove excess exopolysaccharides prior to genomic DNA isolation, the cells were washed twice with equal volumes of sterile, distilled water. Genomic DNA was then isolated from the washed cells using a Wizard Genomic DNA Purification Kit (Promega, Madison, WI) following the kit protocol for Gram-negative bacteria.

### Genome sequencing and assembly

Genome sequencing was performed at the Laboratory for Biotechnology and Bioanalysis at Washington State University on a PacBio RS instrument (Pacific Biosciences, Menlo Park, CA). A small insert library for circular consensus reads was prepared from 5 µg of P101 genomic DNA. The genomic DNA was first fragmented to 1 Kb pieces using 20 shearing cycles at speed code 6 through the small shearing assembly of a Hydroshear Plus (Digilab, Marlborough, MA). The library was then constructed using the DNA Template Prep Kit 2.0 (250 bp- <3 kb) (Pacific Biosciences, Menlo Park, CA). Two large insert (10 Kb) libraries for continuous long reads (CLR) were also prepared. For one library, 10 µg of genomic DNA was sheared using 20 shearing cycles at speed code 11 through the large shearing assembly of a Hydroshear Plus. The second library was prepared with 5 µg of genomic DNA that was fragmented by passing the DNA twice through a g-TUBE (Covaris, Woburn, MA) at 6,000 x g in a microcentrifuge. Both large libraries were prepared using DNA Template Prep Kit 2.0 (3-10 Kb) (Pacific Biosciences). The resulting libraries were bound to the C2 DNA polymerase (Pacific Biosciences) and loaded into the SMRT cell (Pacific Biosciences) zero mode waveguides by diffusion (small libraries and first large library) or with mag-bead assistance (second large library). The prepared libraries were loaded on a total of 16 SMRT cells. The four SMRT cells that contained the small insert libraries were observed with two 55 minute movies while the 12 SMRT cells with large libraries were observed with a single 120 minute movie. Pre-filtering, there was 1.5 Gbp of data in 1.2 million reads with an average read length of 1,244 bp and read quality of 0.284. After filtering to remove any reads shorter than 100 bp or below the minimum accuracy of 0.8, 0.96 Gbp of data remained and consisted of 287,709 reads with an average quality of 0.857 and an average read length of 3,323 bp.

The raw data from the 16 SMRT cells were assembled using the HGAP protocol of the SMRT Analysis v2.0.0 software (Pacific Biosciences). The standard bacterial HGAP assembly protocol with an expected genome size of 5.0 Mb was used. The same protocol was also used to assemble the data from 12 SMRT cells, which excluded four CLR SMRT cells run under instrument software v1.3.0, due to concerns of artifacts in the assembly based on how the quality scores were handled by that version of the software. The 20 contigs from the 16 SMRT cell assembly were used as the base set of contigs. The largest contig was 1.7 Mbp in length and the average coverage for all the contigs was 131× with an N50 of 591,864 bp. The 12 SMRT cell contig set was essentially the same, but there were 28 contigs with an N50 of 3,479,841 bp (also the length of the longest contig). The contigs were mapped to the P101 optical map. This allowed the contigs to be ordered and for overlapping regions to be joined together. Primer pairs for regions throughout the genome assembly were generated and used to verify the assembly using GoTaq Polymerase (Promega) according to the manufacturer’s protocol and 50 ng of P101 genomic DNA, which had an annealing temperature of 52°C and an extension of 1 m. Sequencing was completed for both strands of the PCR amplicons using the same primers used for amplification of the fragments. The assembled chromosome and sequences from the PCR products were aligned with Bioedit (Ibis Biosciences, Carlsbad, CA).

### Genome annotation

The submission file for GenBank was prepared using Sequin [46]. The genome sequence was submitted to GenBank and annotated with the NCBI Prokaryotic Genome Annotation Pipeline [44].

## Genome properties

The genome of *E. cloacae* P101 has one circular chromosome of 5,369,929 bp (Table 3). The average G+C content for the genome is 54.4% (Table 3). There are 100 tRNA genes and 8 rRNA operons, each consisting of a 16S, 23S, and 5S rRNA gene. There are 5,164 predicted protein-coding regions and 29 pseudogenes in the genome. A total of 4,419 genes (83.6%) have been assigned a predicted function while the remainders have been designated as hypothetical proteins (Table 3). The numbers of genes assigned to each COG functional category are listed in Table 4. Of the annotated genes, 19.6% were not assigned to a COG or are of unknown function.

**Table 3:** P101 Genome Statistics

Attribute	Value	% of total^a^
Genome size (bp)	5,369,929	100%
DNA coding region (bp)	4,773,116	88.89%
DNA G+C content (bp)	2,920,174	54.38%
Number of replicons	1	
Extrachromosomal elements	0	
Total genes^b^	5,289	100%
tRNA genes	100	1.89%
rRNA operons	8	
Protein-coding regions	5,164	97.64%
Pseudo genes	29	0.55%
Genes with function prediction	4,419	83.55%
Genes in paralog clusters	3,903	73.79%
Genes assigned to COGs	4,086	77.25%
Genes assigned Pfam domains	4,474	84.59%
Genes with signal peptides	498	9.42%
Genes with transmembrane helices	1,213	22.93%
CRISPR repeats	2	

^a^ The total is based on either the total number of base pairs or the total number of genes in the genome

^b^ Includes the tRNA genes and pseudogenes

**Table 4:** Number of genes associated with the general COG functional categories

Code	Value	%age^a^	Description
J	195	3.8	Translation, ribosomal structure and biogenesis
A	1	0.0	RNA processing and modification
K	432	8.4	Transcription
L	209	4.0	Replication, recombination and repair
B	0	0.0	Chromatin structure and dynamics
D	35	0.7	Cell cycle control, cell division, chromosome partitioning
Y	0	0.0	Nuclear structure
V	56	1.1	Defense mechanisms
T	240	4.6	Signal transduction mechanisms
M	259	5.0	Cell wall/membrane/envelope biogenesis
N	159	3.1	Cell motility
Z	0	0.0	Cytoskeleton
W	0	0.0	Extracellular structures
U	141	2.7	Intracellular trafficking, secretion, and vesicular transport
O	145	2.8	Posttranslational modification, protein turnover, chaperones
C	234	4.5	Energy production and conversion
G	445	8.6	Carbohydrate transport and metabolism
E	385	7.5	Amino acid transport and metabolism
F	88	1.7	Nucleotide transport and metabolism
H	167	3.2	Coenzyme transport and metabolism
I	121	2.3	Lipid transport and metabolism
P	234	4.5	Inorganic ion transport and metabolism
Q	90	1.7	Secondary metabolites biosynthesis, transport and catabolism
R	515	10.0	General function prediction only
S	428	8.3	Function unknown
-	343	11.3	Not in COGs

^a^ The total is based on the total number of protein coding genes in the entire annotated genome
